# Supplementary material for: Exploring confidence development in interprofessional teams: A pre-post analysis of a health and social care education module
Source: PLoS One. 2025 Jul 15;20(7):e0327640. doi: 10.1371/journal.pone.0327640 (PMC12262864; doi:10.1371/journal.pone.0327640)
Supplement: Attachment — Submitted filename: Revision of PONE-D-24-55482.docx [file pone.0327640.s003.docx]

**Revision of PONE-D-24-55482**

The manuscript presents a pre-post study examining the impact of a 6-week academic interprofessional education (IPE) module on students’ self-perceived confidence in interprofessional teamwork. After reviewing the study with PLOSONE revision criteria, I recommend that it is accepted with minor to moderate revisions.

***Criteria 1. The study presents the results of original research***

The study meets the criterion. The manuscript presents original findings derived from a pre-post evaluation of an academic IPE module. The intervention and evaluation context are clearly novel within the scope of higher education in health and social care.

***Criteria 2. Results reported have not been published elsewhere***

The study meets the criterion. There is no evidence of prior publication of these results. The authors should confirm in their cover letter or acknowledgments whether any parts of the dataset were presented in abstracts or institutional reports.

***Criteria 3. Experiments, statistics, and analyses are performed to a high standard and are described in sufficient detail.***

The study partially meets the criterion. The statistical methods used are appropriate. However, the manuscript lacks (a) a priori power analysis to justify the sample size, (b) a more detailed breakdown of how missing data and potential biases (e.g., social desirability) were statistically treated or tested, (c) the psychometric performance of the instrument within the current sample (e.g., Cronbach’s alpha reported for this sample), (d) add justification for sample size (or acknowledge it as a limitation), (e) and clearly describe any tests for assumptions of normality, and (f) reiterate scale reliability for current dataset.

***Criteria 4. Conclusions are supported by the data and presented appropriately.***

The study partially meets the criterion. The findings of increased confidence are well supported by the data. However, the lack of a control group significantly limits causal claims, and while overconfidence and social desirability bias are mentioned, they are not controlled for or empirically explored. The authors should be more cautious in their wording, especially when implying the intervention caused the improvement. They should clarify that changes in self-perception cannot be directly equated with behavioral changes or clinical performance. The lack of inclusion of qualitative findings should also be discussed as they could have strengthened the results of the study.

***Criteria 5. The article is intelligible and written in standard English***

The study meets the criterion. The manuscript is well-written, coherent, and follows academic conventions. However, some redundancy could be reduced (e.g., lengthy background on IPE theory and institutional module structure),

***Criteria 6. The research meets ethical standards and is ethically sound***

The study meets the criterion. Excellent attention to ethical clarity. Consider stating explicitly that the study adhered to the Declaration of Helsinki or relevant national/international standards.

***Criteria 7. The article adheres to reporting guidelines and community data availability standards***

The study partially meets the criterion. Authors should include supplementary material with the reporting guideline or checklist they used (e.g., TREND), and mention it in their methods section. Authors state that all data are fully available without restriction. If not already provided, consider uploading the dataset and questionnaire items as supplementary material or to a repository (e.g., OSF or Figshare) to enhance reproducibility.
