# Supplementary material for: Exploring confidence development in interprofessional teams: A pre-post analysis of a health and social care education module
Source: PLoS One. 2025 Jul 15;20(7):e0327640. doi: 10.1371/journal.pone.0327640 (PMC12262864; doi:10.1371/journal.pone.0327640)
Supplement: Attachment — Submitted filename: Response to Reviewers.docx [file pone.0327640.s006.docx]

**Response to Editor/Email**

| **Editors' comments – Email** | **Actioned/Comments** |
| --- | --- |
| Please ensure that your manuscript meets PLOS ONE's style requirements, including those for file naming. | **Actioned:** Yes, the guidelines were followed. |
| Please provide a complete Data Availability Statement in the submission form, ensuring you include all necessary access information or a reason for why you are unable to make your data freely accessible. If your research concerns only data provided within your submission, please write "All data are in the manuscript and/or supporting information files" as your Data Availability Statement. | **Actioned: Y**es |
| Please include a separate caption for figure 1 in your manuscript. | **Actioned: Y**es. Included just above the reverence list. |
| Please include captions for your Supporting Information files at the end of your manuscript, and update any in-text citations to match accordingly. Please see our Supporting Information guidelines for more information: <http://journals.plos.org/plosone/s/supporting-information> . | **Actioned: Y**es. Included just above the reverence list. |
| We note that there is identifying data in the Supporting Information file < Data File for PLOSOne Submission.xls>. | **Actioned: Y**es |
| We note that this data set consists of interview transcripts. Can you please confirm that all participants gave consent for interview transcript to be published? | **Actioned:** All open text comments were deleted from the data file as they were not included in the paper. Participants provided consent for these comments, which were approved as part of the survey process. The interview transcripts, which constitute the dataset, were used with participant consent. No potentially identifying information is included in the transcripts. Please confirm if further details are needed |

**Response to Reviewers**

| **Reviewer & Editors comments on original document** | **Actioned/Comments** |
| --- | --- |
| **Reviewer 1** | |
| The “Introduction” should be modified, rearranged and divided into some paragraphs based on scientific writing. Paragraph 1 needs to incorporate brief yet comprehensive introduction of Confidence Development in Inter professional Teams and the main question of the research work. | **Actioned:** IN the background section.  To clarify, the purpose of our introduction is to clearly present the context of our study highlighting the importance of IPE in preparing students for collaborative practice. The introduction explains why this research is necessary identifying the gap in literature on the impact of IPE on students self-perceived confidence. Therefore, the introduction provides a concise overview of the key concepts and established the rationale for assessing the student's confidence in this area.  The background section is intended for a more in-depth exploration of the theoretical and empirical foundations related to confidence, IPE and self-efficacy. IT really sets the stage by discussing relevant theories and synthesises past literature to justify the study. The background also emphasises the significance of measure students' confidence in their ability collaborate effectively in interpro3ssional setting...thus we added in a sentence in the background section. Also, the reason why the main question of this research work is at the ed of the background section. |
| The author(s) should modify relevant key words based on MESH. The “Methodology” should be modified, rearranged and divided into some paragraphs based on scientific writing. this section should mention the phases of study very clearly and justify the usage of any statistical tools and approaches one by one in this regard. | We appreciate Reviewer 1 feedback and suggestion to modify the keywords based on MESH terms. However, we have chosen to retain the original keywords as they align with the focus of our study and are consistent with the terminology typically used in the field of IPE. We acknowledge the MESH terms are valuable for indexing and searchability, but we believe the selected keywords are most appropriate for this study's specific context and the audience.  Methodology – we respectfully disagree with the suggestion to restructure and divide it into additional paragraphs. While we understand the need for clearer justification of statistical tools, we feel the current layout and flow aligns with PLOS ONEs established guidance. We have ensured the methodology follows the journals' structure and the statistical tools and approaches used are adequately justified within the context of our study. PLOS ONEs guidance to authors allows for flexibility in methodology presentation and we believe our approach ensures clarity and flow without unnecessary division of content. Moreover, other reviewers have appreciated the structure and organisation and the flow of the article. |
| It is not mentioned how and by which statistical test the validity and reliability of questionnaire were examined | **Actioned:** We have included a reference article which has the details of the statistical tests used for validity and reliability |
| The authors should mention and justify why The sample sizes across different professional groups are unequal, which could reduce the statistical power of subgroup analyses, | **Actioned:** Justification included in the ‘study sample’ paragraph. |
| The authors should justify the low response rate (26% for post-test) that introduces nonresponse bias, meaning the results may not be representative of the entire student cohort. | **Actioned:** Justification included in the ‘response rate’ paragraph and in the limitation section. |
| In samples Among the 80 students 86% identified as female, Dose Gender have any influence on the result of the research? please mention it. | **Actioned:** Included in the limitations section |
| The authors should mention how they control the confounding factors in this research like time, prior experience, different motivation levels of student, etc. | **Actioned:** Included in the limitations section. |
| The "Result", "discussion" and "Conclusion" should be written based on scientific writing approach and also arranged based on the main and specific objectives of the research work. | Thank you for the feedback. Similar to our response to Reviewer 1 second feedback - we have followed the PLOS One Guidance to authors. |
| I found it hard to follow the paper, Consistency and coherency of whole manuscript is weak and should be improved. | Thank you for the feedback. Similar to our response to Reviewer 1 second feedback - we have followed the PLOS One Guidance to authors. |
| **Reviewer 2** | |
| The authors should confirm in their cover letter or acknowledgments whether any parts of the dataset were presented in abstracts or institutional reports. | **Actioned:** as a part of the revisions update during submission of the revisions. |
| lacks (a) a priori power analysis to justify the sample size, | **Actioned:** Included in the limitations section. |
| (b) a more detailed breakdown of how missing data and potential biases (e.g., social desirability) were statistically treated or tested, | **Actioned:** Expanded in the ‘missing data’ section, and social desirability bias in the discussion section. |
| (c) the psychometric performance of the instrument within the current sample (e.g., Cronbach’s alpha reported for this sample), | We appreciate the reviewer's suggestion to report the Cronback’s alpha for the IPE ABC for the current sample. However, as this study is primarily aimed to evaluate the impact of our intervention on student confidence, psychometric testing of the scale was beyond the remit of this research. While we acknowledge the importance of assessing the reliability of this instrument, especially in new populations, this study relied on existing psychometric evidence from prior research (Blumenthal, 2022), where the IPE ABC scale has been shown to have ‘very good’ internal consistency (Alpha = 0.93). |
| (d) add justification for sample size (or acknowledge it as a limitation) | **Actioned:** Acknowledged in the limitation. |
| , (e) and clearly describe any tests for assumptions of normality, | **Actioned;** Added detail re tests used and significance value – under ‘questionnaire analysis confidence’ in the methods section |
| (f) reiterate scale reliability for current dataset. | We appreciate the reviewer's suggestion to report the Cronback’s alpha for the IPE ABC for the current sample. However, as this study is primarily aimed to evaluate the impact of our intervention on student confidence, psychometric testing of the scale was beyond the remit of this research. While we acknowledge the importance of assessing the reliability of this instrument, especially in new populations, this study relied on existing psychometric evidence from prior research (Blumenthal, 2022), where the IPE ABC scale has been shown to have ‘very good’ internal consistency (Alpha = 0.93). |
| However, the lack of a control group significantly limits causal claims, and while overconfidence and social desirability bias are mentioned, they are not controlled for or empirically explored. The authors should be more cautious in their wording, especially when implying the intervention caused the improvement. They should clarify that changes in self-perception cannot be directly equated with behavioral changes or clinical performance. The lack of inclusion of qualitative findings should also be discussed as they could have strengthened the results of the study. | **Actioned:**  **More cautious in the wording:** softened abstract and paper conclusion; This study contributes to the existing literature by reinforcing the positive influence of IPE interventions on behaviours that support collaborative practice, however these results should be interpreted with the study limitations in mind  Also highlighted not generalisable to practice in limitation section   **Lack of control:** We acknowledge the absence of control group as a limitation. However, we would like to respectfully note that the primary aim of this study was to explore changes in students perceived self-confidence following an educational intervention in a real-world academic setting. By introducing a control group, where some students would be withheld from participating in a compulsory timetabled learning class would not have been ethical or practically appropriate in this context. Given the nature of integrated IPE activities all students are expected to participate, and random assignment of control and experimental groups is not feasible. In addition, the use of pre-post design in an academic context is consistent with similar studies in educational research and IPE literature. Nevertheless, we agree that future studies could benefit from the inclusion of control or comparator groups where feasible. This would strengthen causal inference and explore the sustainability of perceived improvements. Therefore, we have added a line in the limitations section on the absence of control group. |
| However, some redundancy could be reduced (e.g., lengthy background on IPE theory and institutional module structure), | **Actioned:** Amended introduction and a bit of background to reduce information in these sections |
| Consider stating explicitly that the study adhered to the Declaration of Helsinki or relevant national/international standards. | **Actioned:** Yes, in the cover letter for ethics. |
| Authors should include supplementary material with the reporting guideline or checklist they used (e.g., TREND), and mention it in their methods section. Authors state that all data are fully available without restriction. If not already provided, consider uploading the dataset and questionnaire items as supplementary material or to a repository (e.g., OSF or Figshare) to enhance reproducibility. | **Actioned:** Included as supplementary material. However, we did not map to TREND checklist as it was deemed inappropriate. TREND checklist is primarily intended for non-randomized public health or clinical trials and is not the best fit for educational intervention study conducted in an academic setting with a pre-post design. For this study – a quantitative, nonrandomised, pre-post study evaluating an education intervention the most appropriate checklist is STROBE. Of the three different STROKE checklists, the closest one was the cohort design, and it is widely accepted in educational research. |
| **Reviewer 3** | |
| Abstract - Strengths: Clearly presents the aim, methods, results, and conclusions. Inclusion of effect sizes and significance values is commendable. Recommendations: Avoid causative language like “suggesting the IPE intervention had a positive impact” unless supported by stronger experimental controls. Suggested revision: “indicating an association between the IPE intervention and increased self-perceived confidence.” | **Actioned:** amended abstract and conclusion |
| Introduction - Strengths: Comprehensive overview of IPC and IPE significance, backed by strong references. Suggestions: The introduction would benefit from sharper articulation of the knowledge gap. It currently blends motivation with background without clearly stating what this study adds. Suggested addition: “Despite widespread endorsement of IPE, few studies have evaluated validated tools in pre-registration academic settings using robust pre-post designs.” | **Actioned:** Added recommendation to end of introduction section |
| Background - Strengths: Theoretical framework (self-efficacy, expectancy-value theory) is well-integrated. Suggestions: The background could be more concise. Some of the historical institutional information may be more appropriate for supplementary material unless critical for methodology. | **Actioned:** shortened the section as requested. |
| Methods - Strengths: Methodology is described in strong detail with ethical approvals, sampling, instruments, and analysis procedures clearly stated. Use of a validated scale (IPE-ABC) and description of subscales adds robustness. Recommendations: Sampling bias is likely due to low response rates (26% post-test). This should be acknowledged more prominently in the methods or limitations. Data matching by alias codes is creative, but the risk of participant error or dropouts affecting match quality should be noted. | **Actioned:** Limitations amended to reflect this request. |
| Results - Strengths: Clear presentation of results across subscales with robust statistical analysis (paired t-tests, Wilcoxon tests, ANOVA). Use of both Cohen’s d and Pearson’s r for effect size is a good practice. Suggestions: Use of negative values for Cohen’s d (e.g., d = –0.888) is unconventional. Since effect size direction is not meaningful in pre-post designs where direction is known, report absolute values for clarity. Discuss implications of high baseline scores and possible ceiling effects—several participants were already “very confident” pre-module. Tables are comprehensive but visual aids (e.g., bar charts or boxplots) could help illustrate change distributions. | **Actioned:** Cohen’s d values amended as requested.  Ceiling effect covered in limitations.  **Tables & Visual aids:** We agree that visual aids can be valuable in illustrating the distribution and change scores and enhance interpretability. However, in this study we aimed to present a clear and concise summary of the pre-post changes using appropriate statistical measures within a tabular form. This format was chosen to align with the journals formatting expectations and to ensure readability and precision of statistical outcomes. We also considered the fact that the primary aim of this study was not to analyse distributional patterns in depth, but rather than assess the overall changes in self-reported confidence using IPEABC scores. The tables already reflect both central tendency and variability including effect sizes. Nonetheless we appreciate the reviewers comment and will explore incorporating visual representation such as boxplots in future dissemination formats i.e. conference representation or subsequent publications that requires more illustrative approach. |
| Discussion - Strengths: Strong integration with previous studies Addresses psychological theories (self-efficacy, overconfidence bias, SDRB), which adds analytical depth. Suggestions: The narrative is overly descriptive in places; streamline and focus on key implications (e.g., the need for continuous confidence calibration, not just confidence building). Provide practical recommendations for educators: how might one modify IPE modules to address overconfidence or SDRB? Highlight potential Type II error risk more explicitly in subgroup comparisons (e.g., small male sample). | **Actioned:**  Continuous confidence calibration: included a sentence in the discussion.  Practical recommendations to educators: included a paragraph in the discussion.  Type 2 error: Included two sentences in the discussion. |
| Limitations - Strengths: Thoughtfully acknowledges low response rate, sample imbalance, and SDRB. Plans for longitudinal follow-up are commendable.  Additional Suggestions: Add a statement on generalizability—results may not apply to students in non-academic or clinical IPE settings. Clarify whether the low male participation is reflective of program enrollment or introduces potential gender bias in outcomes. | **Actioned:** A few sentences spread out in the limitations section. |
| Conclusion - Strengths: Accurately reflects the findings without overstating them. Reinforces the contribution of validated tools to IPE evaluation. Recommendation: Emphasize the need for future research on the translation of academic confidence into clinical settings, especially during transition to practice. | **Actioned:** added statement to conclusion and limitations |
| Technical Aspects & PLOS ONE Fit Fit for Journal: The topic aligns well with PLOS ONE’s focus on interdisciplinary, educational, and public health research. Writing & Structure: Language is professional and generally clear, though some sections (e.g., Background, Discussion) are verbose and would benefit from editing for brevity. | **Actioned:** Thank you. We have addressed the background and discussion accordingly. |
|  |  |
| **Reviewer 4** |  |
| I found the paper very interesting and well written and organized. I would like some more graphics but the information was still all there. Just a few small suggestions - to include a description of the acronym IPE in the abstract and I wasn't sure I completely understood the key of table 4, in particular: very confidence levels of confidence sounded odd. Also in the results for example it was stated that the confidence level in males was higher but only provided the value of confidence for males. But they are very minor comments. Overall I found the paper very interesting and well written. | Thank you kindly for your feedback. |
