# Supplementary material for: Exploring confidence development in interprofessional teams: A pre-post analysis of a health and social care education module
Source: PLoS One. 2025 Jul 15;20(7):e0327640. doi: 10.1371/journal.pone.0327640 (PMC12262864; doi:10.1371/journal.pone.0327640)
Supplement: Attachment — Submitted filename: Revision of PONE-D-24-55482R1.docx [file pone.0327640.s005.docx]

**Revision of PONE-D-24-55482R1**

This manuscript presents an original empirical study that evaluates the impact of an interprofessional education module on the self-perceived confidence of students in health sciences and social services. The focus is relevant and timely, addressing a gap in literature using a validated tool and appropriate statistical analyses. The work is suitable for a journal like PLOS ONE, with some minor revisions.

**Criterion 1. The study presents the results of original research**

The manuscript addresses a relevant issue in the training of health and social care professionals: the development of confidence in interprofessional contexts. The study's approach is novel in the field, particularly in its use of a validated scale in a pre-post design with undergraduate students. No duplications or prior publications using the same dataset were identified. In this regard, the study satisfactorily meets the originality criterion.

**Criterion 2. Results reported have not been published elsewhere**

The authors state that the results have not been previously published, and a review of the content reveals no signs of redundancy or overlapping with prior studies. The analysis uses a recently validated tool, which supports the study’s originality.

**Criterion 3. Experiments, statistics, and analyses are performed to a high standard and are described in sufficient detail**

The methodology is clear and appropriate for the research question. A pre-post design is employed which, although lacking a control group, is reasonable within the educational context studied. The statistical methods are well justified, distinguishing between parametric and non-parametric tests according to data distribution. Confidence intervals and effect sizes (Cohen’s d and Pearson’s r) are reported, enabling interpretation of the magnitude of change. Nonetheless, this section could be strengthened by including sample size justification, either through a post hoc power analysis or a more explicit discussion of the feasibility and limitations of convenience sampling. Additionally, a brief mention of the potential influence of the data imputation method (mean) on the results would be helpful, although the percentage of missing cells was low.

**Criterion 4. Conclusions are supported by the data and presented appropriately**

The conclusions are directly derived from the results and reflect a significant increase in students’ perceived self-confidence following the intervention. However, greater caution is recommended in presenting “time” as the sole significant factor, considering the small size of subgroups and the potential for Type II errors. It would also be advisable to move part of the reflection on social desirability bias and overconfidence from the discussion into the conclusions, acknowledging their possible effects on the interpretation of findings.

**Criterion 5. The article is intelligible and written in standard English**

The manuscript is written in clear English but could benefit from language editing to improve fluency, eliminate repetition, and clarify some transitions. While the introduction and discussion are comprehensive, they contain long paragraphs that hinder readability. Breaking up some sections and reviewing terminology consistency is recommended.

**Criterion 6. The research meets ethical standards and is ethically sound**

The manuscript appropriately presents the ethical aspects. Institutional ethics committee approval was obtained, participant confidentiality was respected, and informed consent was adequately ensured. Anonymity and the separation of roles between educators and evaluators enhance the integrity of the study.

**Criterion 7. The article adheres to reporting guidelines and community data availability standards**

The study is reported according to the STROBE guidelines for observational studies. Data availability is clearly stated, and the authors confirm that the underlying data are fully accessible, aligning with the journal’s open-access policies.

**Additional Recommendations**

In the introduction, the section on interprofessional education could be slightly shortened to avoid redundancy. It is suggested to define the concept of confidence more concisely at the beginning of the text. In the methods section, it would be pertinent to justify the use of a four-point Likert scale and clarify whether the module was mandatory or elective. In the results section, some tables could be summarized or moved to supplementary material. In the discussion, literature integration is well done, but practical implications could be further developed, for example, by proposing strategies to calibrate confidence, such as structured reflection or peer assessment. In the limitations section, it would be appropriate to add a note on potential nonresponse bias and the inability to generalize findings to clinical settings. Finally, the conclusion’s language should be moderated, avoiding direct causal claims that the study design does not support.
