# Supplementary material for: Exploring confidence development in interprofessional teams: A pre-post analysis of a health and social care education module
Source: PLoS One. 2025 Jul 15;20(7):e0327640. doi: 10.1371/journal.pone.0327640 (PMC12262864; doi:10.1371/journal.pone.0327640)
Supplement: Attachment — Submitted filename: Response_to_Reviewers_auresp_2.docx [file pone.0327640.s007.docx]

| **Reviewers Comments** | **Action** |
| --- | --- |
| The language around causality in parts of the discussion and abstract could still be further softened to emphasize associative, not causal, findings. | **Actioned:** Conclusion in abstract amended to reflect the potential impact of social desirability response bias, overconfidence bias and small subgroups on findings and that interpretation of results needs to reflect these considerations. Lines 57-62 |
| Consider changing phrasing from “the module had a positive impact” to “the module was associated with increased confidence,” to maintain alignment with observational design. | **Actioned:** Changed the phrase as recommended at the start of the discussion section, also softened it in the abstract. |
| Discussion  Though improved, some speculative comments on “real-world applicability” (e.g., bridging to practice) could benefit from hedging language due to the study’s academic-only context. | **Actioned:** At two places. Included 2 sentences at the very end of the discussion section. Also rephrased the language in the conclusion section. |
| Conclusion  Consider ending with a more actionable statement, such as: “Validated self-report tools such as IPE-ABC can support curriculum developers in measuring and adjusting pre-registration IPE design.” | **Actioned:** Amended slightly and added to a sentence to the conclusion section reflecting the suggestion made. |
| **Reviewer 2** |  |
| Nonetheless, this section could be strengthened by including sample size justification, either through a post hoc power analysis or a more explicit discussion of the feasibility and limitations of convenience sampling. | **Actioned:** Feedback addressed by inclusion of two sentences in the participants section (under methods). |
| Additionally, a brief mention of the potential influence of the data imputation method (mean) on the results would be helpful, although the percentage of missing cells was low. | **Actioned:** The last line was rewritten in the data analysis section in methods, to include the potential influence of the data imputation method. |
| However, greater caution is recommended in presenting “time” as the sole significant factor, considering the small size of subgroups and the potential for Type II errors. It would also be advisable to move part of the reflection on social desirability bias and overconfidence from the discussion into the conclusions, acknowledging their possible effects on the interpretation of findings. | **Actioned:** Made a statement relating to impact of SDRB, overconfidence bias and type 11 error |
| The manuscript is written in clear English but could benefit from language editing to improve fluency, eliminate repetition, and clarify some transitions. While the introduction and discussion are comprehensive, they contain long paragraphs that hinder readability. Breaking up some sections and reviewing terminology consistency is recommended. | **Actioned:** We have reviewed the manuscript for paragraph length and have split longer paragraphs into shorter ones and also made it much more concise. This should help with readability and flow. We also reviewed and standardised terminology throughout the manuscript to ensure consistency. |
| In the introduction, the section on interprofessional education could be slightly shortened to avoid redundancy. It is suggested to define the concept of confidence more concisely at the beginning of the text. | **Actioned:** Introduction shortened considerably, based on this and the above comment. Confidence is defined in a much more concise manner. |
| In the methods section, it would be pertinent to justify the use of a four-point Likert scale and clarify whether the module was mandatory or elective. | **Actioned:** First feedback actioned din the Outcome Measures section of the methods. Second feedback in the first line in the methods. |
| In the results section, some tables could be summarized or moved to supplementary material. | **Not Actioned:** Thank you for your suggestion. We appreciate the feedback; however, we believe that retaining the full tables in the main results section is important for ensuring transparency, allowing readers to fully interpret the subgroup analysis for age, gender and professional group. This study aimed to explore the potential influences of these variables on confidence outcomes, presenting the full and complete breakdown supports the aims and aligns with the reporting best practices for observational studies. We therefore prefer to retain the tables in the main manuscript for clarity and completeness. |
| In the discussion, literature integration is well done, but practical implications could be further developed, for example, by proposing strategies to calibrate confidence, such as structured reflection or peer assessment. | **Actioned:** Thank you, we appreciate this suggestion, however we did include some suggestions for structured reflection and safe places in the discussion. However, we agree the premise and added a couple of sentences providing concrete examples in the third paragraph of the discussion. |
| In the limitations section, it would be appropriate to add a note on potential nonresponse bias and the inability to generalize findings to clinical settings. | **Actioned:** In the first paragraph of the limitations section. |
| Finally, the conclusion’s language should be moderated, avoiding direct causal claims that the study design does not support. | **Actioned:** The causal claims are actioned |
